# Out-of-Pocket Expenditure (OOPE) Among COVID-19 Patients by Insurance Status in a Quaternary Hospital in Karnataka, India

**DOI:** 10.3390/ijerph22081289

**Published:** 2025-08-18

**Authors:** Rajesh Kamath, Chris Sebastian, Varshini R. Jayapriya, Siddhartha Sankar Acharya, Ashok Kamat, Helmut Brand, Reshma Maria Cocess D’Souza, Prajwal Salins, Aswin Sugunan, Sagarika Kamath, Sangita G. Kamath, Sanjay B. Kini

**Affiliations:** 1 Department of Healthcare and Hospital Management, Prasanna School of Public Health, Manipal Academy of Higher Education, Manipal 576104, India; rajesh.kamath@manipal.edu (R.K.); chris.psphmpl2023@learner.manipal.edu (C.S.); varshini.psphmpl2023@learner.manipal.edu (V.R.J.); 2 Department of Medical Administration, Tata Memorial Hospital, Mumbai 400012, India; siddhartha.acharya18@gmail.com; 3 Department of Psychiatry Nursing, Vijaya College of Nursing Sciences, Belagavi 590010, India; kamat944@gmail.com; 4 Department of International Health, Care and Public Health Research Institute—CAPHRI, Faculty of Health, Medicine and Life Sciences, Maastricht University, 6200 MD Maastricht, The Netherlands; helmut.brand@maastrichtuniversity.nl (H.B.); dr.sagarikarkamath@gmail.com (S.K.); 5 Department of Medical Laboratory Technology, Manipal College of Health Professions, Manipal Academy of Higher Education, Manipal 576104, India; reshma.dsouza@manipal.edu (R.M.C.D.); prajwal.salins@manipal.edu (P.S.); 6 Department of Hospital Administration, Yenepoya (Deemed to Be University), Mangalore 574153, India; aswinsugunan@yenepoya.edu.in; 7 Department of Pharmacology, Kasturba Medical College, Manipal, Manipal Academy of Higher Education, Manipal 576104, India; 8 Department of Community Medicine, Kasturba Medical College, Manipal, Manipal Academy of Higher Education, Manipal 576104, India

**Keywords:** out-of-pocket expenditure, COVID-19, AB-PMJAY, private health insurance, uninsured patients

## Abstract

Out-of-pocket expenditure (OOPE) comprises 62% of national health expenditure in India. This heavy reliance on direct payments has engendered economic vulnerability and catastrophic financial pressures (typically defined as out-of-pocket spending exceeding a certain threshold of household income, leading to financial hardship) on households in a country where public health spending remains below targeted levels. The onset of the COVID-19 pandemic intensified these financial hardships further, as both total healthcare spending and OOPE experienced significant escalations due to the increased need for emergency care, vaccination efforts, and expanded health infrastructure. A retrospective, single-center study was conducted using data from COVID-19 patients admitted between June 2020 and June 2022. Patient data were collected from the Medical Records, IT, and Finance departments. A validated proforma was used for data extraction. Descriptive statistics were calculated, and the Shapiro–Wilk test was applied to assess normality of billing and OOPE data. Patients were stratified into three groups based on their insurance status, allowing for comparative analysis of OOPE percentages and absolute expenditures. The 2715 COVID-19 patients were categorized into three groups according to their health financing: those covered under AB-PMJAY (42.76%), private health insurance (22.16%), and the uninsured (35%). While the median billing amounts were comparable across these groups (ranging between INR 85,000 and INR 90,000), a substantial disparity was observed in terms of financial burden. All patients covered under AB-PMJAY incurred no OOPE, whereas privately insured patients had a median OOPE that constituted approximately 21% of their total billing amounts, with significant variability among different insurers. The uninsured group represented 35% of the cases and experienced the highest median OOPE, indicating substantial financial risk. The COVID-19 pandemic has revealed critical gaps in India’s health financing framework. This study emphasizes the strong financial protection provided by AB-PMJAY, while also exposing the limitations of private health insurance in shielding patients from substantial healthcare costs. As the country progresses toward universal health coverage, there is a pressing need to expand public health insurance schemes that are inclusive, equitable, and effectively implemented. Additionally, strengthening regulation and accountability in the private insurance sector is essential. The study findings reinforce that AB-PMJAY has been highly successful in reducing OOPE and enhancing financial risk protection. Although private insurance reduced OOPE, patients still faced considerable expenses. The stark difference in OOPE of 100% for uninsured patients, 21.16% for privately insured, and 0% for AB-PMJAY beneficiaries underscores the importance of further expanding AB-PMJAY to reach more vulnerable populations.

## 1. Introduction

Out-of-pocket expenditure (OOPE) constitutes approximately 62% of total health expenditure (THE) in India. It remains a critical challenge within the healthcare system, placing significant financial pressure on households, often reaching levels considered catastrophic health expenditure. The predominance of the direct payment model is compounded by public health expenditure consistently falling short of targeted benchmarks. This has significantly increased economic vulnerability, particularly among low-income populations. This heavy reliance on OOPE disproportionately affects low-income individuals, deepening income inequality and perpetuating a cycle of medical poverty. Increasing public health spending to 3% of the gross domestic product (GDP) has the potential to reduce OOPE to 30% of total health expenditure. Although successive governments have consistently pledged to raise public health expenditure to this level, these commitments have not materialized, with current allocations remaining at only 2.2% of GDP [1,2,3].

India is ranked third in Southeast Asia and 67th out of 189 nations for per capita healthcare OOPE, adjusted for purchasing power parity (PPP) [4,5]. Notably, 49% of households that accessed hospitalization or outpatient services encountered catastrophic health expenditure, and 15% were pushed below the poverty line due to OOPE [6]. THE in 2019 was INR 2.73 lakh crores (USD 3.24 billion) with OOPE at 1.31 lakh crores (USD 1.55 billion) (48.2% of THE). THE in 2022 rose to INR 4.72 lakh crores (USD 6.3 billion) with OOPE at 2.59 lakh crores (USD 3.48 billion) (55% of THE) [7]. The COVID-19 pandemic significantly impacted healthcare spending and individual financial burdens. Between 2019 and 2022, THE in India increased at a compound annual growth rate (CAGR) of approximately 20.06%. In comparison, OOPE exhibited a higher CAGR of approximately 25.25% over the same period. This differential growth indicates a disproportionate escalation in the financial burden on individuals, with a growing share of healthcare costs being met directly by households rather than through pooled or public financing mechanisms. The increase in total healthcare expenditure of 72.8% reflects the substantial financial demands of managing the pandemic, including costs for emergency care, vaccinations and enhanced health infrastructure. OOPE nearly doubled, reflecting a 97.7% increase with a CAGR of 25.25%.

In September 2018, the Government of India launched the Ayushman Bharat Pradhan Mantri Jan Arogya Yojana (AB-PMJAY), a publicly funded health insurance designed to provide financial protection to the bottom 40 percent of the population. The AB-PMJAY was intended to cover over 100 million families or approximately 500 million individuals. However, awareness of the scheme varies significantly across regions, with rural Jammu reporting an awareness rate as low as 28% compared to 77% in rural Tamil Nadu. These regional disparities underscore the challenges in achieving universal health coverage and highlight the critical need for enhanced awareness of and improved accessibility to the Publicly Funded Health Insurance (PFHI) schemes so that target populations can benefit from the initiative [8,9].

In 2021, it was estimated that approximately 70% of the Indian population had some form of health insurance. Of this, around 50% (700 million individuals) were covered by the AB-PMJAY and various state-specific schemes. Additionally, 20% of the population (280 million people) were covered by social health insurance and private voluntary health insurance. Approximately 30% of the population remained uninsured. The 30% of the population that remains uninsured largely belongs to the ‘missing middle’, individuals who are above the poverty line and thus ineligible for publicly funded schemes like AB-PMJAY, but who also lack access to employer-based or private insurance. In addition to income-based exclusions, challenges such as lack of awareness, administrative barriers, and the absence of required documentation at the point of care further contribute to this coverage gap. Health insurance premiums rose by 40% in 2020, largely driven by an increased effort to secure coverage during the COVID-19 pandemic. The rise in private healthcare utilization and insurance premiums in 2022 can be largely attributed to COVID-19. This upward trend continued in 2022, with premiums rising by 34.2%, compared to a 9.9% increase in 2021. Nevertheless, a substantial portion of the population remains without health insurance, highlighting a critical gap in coverage [10,11].

COVID-19 was declared a global pandemic on 11 March 2020. There were over 753 million confirmed cases and more than 68 million deaths reported globally. India reported 43 million confirmed cases and 500,000 deaths [12,13]. The pandemic imposed a significant financial strain on families across the globe through medical care costs and wage loss. In 2020, the global labor market experienced a 7.3 percentage drop in the employment-to-population ratio. Urban areas were particularly hard-hit, with the unemployment rate going over 20%. The global poverty rate increased by 11%. This economic strain was further reflected in the global Gini coefficient, which increased by more than 0.5 points, indicating a marked rise in income inequality. Notably, 7% of India’s population was pushed into poverty. The global poverty rate increased by 4 percentage points [11,12,14,15,16]. Low-income patients with COVID-19 face substantial financial barriers that critically impact their ability to access timely healthcare. Despite the availability of AB-PMJAY and private health insurance, a segment of the population continues to bear the burden of OOPE. While AB-PMJAY is designed to mitigate the financial impact on low-income households, private insurance often provides only partial coverage for COVID-19-related expenses, leaving many patients with substantial OOPE. This study seeks to compare OOPE for COVID-19 patients during their care at a tertiary care teaching hospital from June 2020 to July 2022. By analyzing spending patterns during this period, the study aims to offer insights that can help improve policies and make healthcare accessible for patients by reducing their financial burden. This study contributes to the limited body of literature that quantifies out-of-pocket expenditure for COVID-19 inpatients across different insurance groups using actual hospital billing data in India. The subsequent sections are organized as follows: Section 2 describes the methodology and study setting, Section 3 presents the key findings, Section 4 discusses these findings in light of the existing literature, and Section 5 concludes with policy implications and limitations.

## 2. Research Methodology

Study Setting: A retrospective study was conducted with data generated from COVID-19 inpatient cases between June 2020 and June 2022, in a tertiary care 2000-bedded teaching hospital in Karnataka state of India. The hospital serves about 1500 inpatients and 2500 outpatients daily. The hospital is accredited by the National Accreditation Board for Hospitals and Healthcare Providers (NABH). All COVID-19 cases except COVID-19 deaths were included in the study. A retrospective single-center study involves the analysis of existing patient data collected at a single institution over a defined period. This design is particularly suitable for assessing patterns such as out-of-pocket expenditure during the COVID-19 pandemic, as it allows for the use of complete hospital billing records and minimizes the need for new data collection during a period of healthcare system disruption.

Study Design: Retrospective single-center study.

Departments Involved:

(1) Medical record department;
(2) IT department;
(3) Finance department.

Inclusion and Exclusion Criteria:

Inclusion Criteria:

(1) COVID-19 inpatients from June 2020 to June 2022;
(2) COVID-19 inpatients with publicly funded health insurance (AB-PMJAY), with private health insurance, and with no insurance.

Exclusion criteria:

(1) COVID-19 deaths were excluded from the analysis due to inconsistencies in billing documentation and challenges in capturing final OOPE values. In many cases, bills were either waived, subsidized, or contested, which could lead to misrepresentation of the actual financial burden. Additionally, ethical and procedural considerations limited access to comprehensive financial records for deceased patients.
(2) Outpatient services were not included in this analysis, as they are generally not covered under AB-PMJAY or standard private health insurance policies in India. The focus of the study was on inpatient financial burden, which forms the core of coverage under publicly funded and most private insurance schemes.

Sample Size: 2715 patients.

Tools used: A validated proforma was used.

Statistical Method: Descriptive statistics, including the mean, median, and the Shapiro–Wilk test for assessing data normality, were employed to analyze and compare the variability in out-of-pocket expenditure (OOPE) across different patient groups, namely AB-PMJAY beneficiaries, individuals with private health insurance, and uninsured patients.

Ethical Consideration: Institutional Ethics Committee (IEC) approval was received. The patient data were anonymized and used only for the purposes of the study.

## 3. Results

This study included 2715 COVID-19 inpatients from June 2020 to June 2022 across three categories: patients with publicly funded health insurance (AB-PMJAYt), with private health insurance, and with no health insurance. Table 1 presents the comprehensive distribution of 2715 cases across various categories: 1161 ABPMJAY cases (42.76 percent), 606 private health insurance cases (22.32 percent), and 948 cases of uninsured patients (34.91 percent). This study was conducted in a quaternary care teaching hospital in Karnataka, which caters to both urban and rural populations.

While the hospital receives a diverse patient mix, the insurance distribution observed in Table 1 may not fully reflect national averages.

## 4. Variation in OOPE Across Various Patient Categories

Table 2 presents the number of cases and the median and mean billing amounts for the selected categories of patients. The median billing amount was INR 126,260 (USD 1694.40) under AB-PMJAY, INR 92,938 (USD 1247.22) under private health insurance, and INR 66,929 (USD 898.18) for uninsured patients. OOPE as an average percentage of the billing amount was zero under AB-PMJAY, 21.16% under private health insurance, and 100% for uninsured patients. The median/mean OOPE was zero for AB-PMJAY, INR 19,728 (USD 264.75) for private health insurance, and INR 66,929 (USD 898.18) for uninsured patients.

Table 3 presents OOPE as a percentage of the billing amount for different private health insurance providers. It provides insights into the financial burden borne by insured patients despite having private health insurance coverage. The total OOPE as a percentage of the billing amount across all insurance providers was 21.16%, meaning that, on average, policyholders had to pay around one-fourth of the billed amount themselves. Sampoorna Suraksha had the highest OOPE percentage (40.26%**),** indicating that policyholders covered by this insurer had to bear a significant portion of medical expenses. TATA AIG General Insurance Co., Ltd. (Mumbai, India) had 0% OOPE, suggesting full coverage for the single case recorded. Universal Sompo General Insurance Co., Ltd. (Mumbai, India) (32.48%) and Star Health and Allied Insurance Co., Ltd. (Mumbai, India) (32.53%) also had high OOPE percentages, highlighting gaps in coverage. Some insurers, like Ecron Acunova Ltd. (Mumbai, India) (0.0%) and Future Generali India Insurance Co., Ltd. (Mumbai, India) (0.28%), had low OOPE percentages, suggesting better financial protection for policyholders.

**Figure 1:**
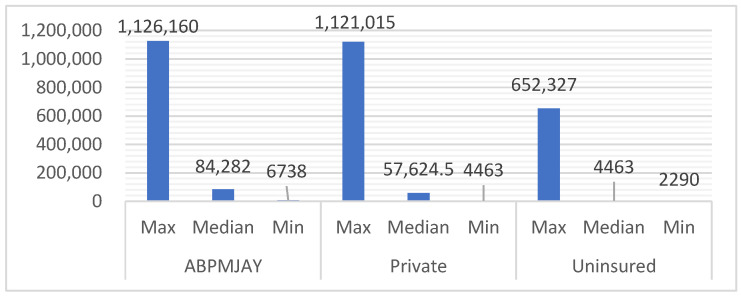
Billing across categories: minimum, maximum, and median.

Figure 1 provides an overview of the billing amounts of COVID-19 patients with publicly funded health insurance (AB-PMJAY), private health insurance, and no health insurance. Under AB-PMJAY, bills ranged from INR 6738 (USD 90) at the lower end to as high as INR 1,126,160 (USD 15,113), with a median of INR 84,282 (USD 1131.06). For those with private insurance, costs varied between INR 4463 (USD 60) and INR 1,121,015 (USD 15,044), with a median bill of INR 57,622 (USD 773.28). Uninsured patients who paid in cash saw bills starting at INR 2290 (USD 31) and going up to INR 652,327 (USD 8754.18), with a median of INR 36,074 (USD 484.11). Since the billing amounts for AB-PMJAY and cash-paying patients did not follow a normal distribution (as confirmed by the Shapiro–Wilk test, *p* < 0.005), the median was used. On the other hand, private insurance bills were normally distributed (*p* > 0.05), meaning the mean is a better measure of central tendency.

## 5. Discussion

There is scarce peer-reviewed literature on OOPE among COVID-19 patients, especially in the Indian context. Our findings are consistent with a previous study that found significantly lower OOPE among individuals covered by public health insurance schemes compared to those with private or no insurance [2]. Similarly, a few studies demonstrated that publicly funded schemes in India were more effective in reducing OOPE than private models [17,18]. Although there is limited research specifically on OOPE during COVID-19 in India, studies have highlighted the broader impact of OOPE and the protective role of health insurance in mitigating financial distress [6,15]. The present study offers critical insights into the OOPE incurred by 2715 COVID-19 patients between June 2020 and June 2022 with publicly funded health insurance (AB-PMJAY), with private health insurance, and with no health insurance in the Infectious Disease Department of a quaternary care teaching hospital in Karnataka state of India. The results highlight significant disparities in OOPE across these categories, pointing to ongoing challenges in healthcare affordability and insurance coverage. A substantial proportion of patients (57.23%) were uninsured, reflecting gaps in health insurance penetration. This aligns with national trends, where a large proportion of the population still lacks adequate financial protection for health emergencies [19].

Although AB-PMJAY is structured to eliminate OOPE by design, our findings provide evidence that this intent translated into practice even during a public health crisis, highlighting successful implementation at the point of care. Notably, 100 percent of AB-PMJAY beneficiaries incurred no OOPE. Individuals with private health insurance incurred lower OOPE than the uninsured. This suggests that, unlike publicly funded health insurance schemes, private insurers may not fully cover all medical expenses of COVID-19 patients [17,18]. The findings reveal a disparity in financial burden among the different patient groups. Patients with private health insurance had an average OOPE of INR 19,728 (USD 264.75), which constituted 21.16% of the billed amount. The highest burden was observed among uninsured patients, who bore 100% of their medical costs, with a median OOPE of INR 66,929 (USD 844.50). These results reinforce existing research indicating that lack of insurance contributes significantly to financial hardship among COVID patients [20]. The study confirms that AB-PMJAY serves as a strong financial safeguard, effectively eliminating OOPE for its beneficiaries and enabling access to essential healthcare without monetary strain. This supports existing government findings that highlight the scheme’s success in curbing OOPE for economically vulnerable populations [3]. AB-PMJAY beneficiaries constituted 42.76% of the total study sample. In urban trauma centers, barriers such as lack of awareness, absence of necessary documents at the time of admission, and administrative hurdles continue to prevent eligible patients from utilizing the scheme effectively [5,21].

While private health insurance does reduce OOPE to some extent, the observed variation among insurance providers points to significant inconsistencies in coverage and financial protection. Only 2.86% of private health insurance patients experienced zero OOPE, indicating the limitations of existing private insurance models in India. While private insurance is often considered a premium alternative, the data highlight its inconsistent performance in real-world scenarios. In this study, privately insured patients incurred an average OOPE of 21.16% of the total billed amount. This residual burden is likely due to factors such as policy exclusions, co-payments, sub-limits, and delays or denials in claim processing. For instance, certain private insurers like Universal Sompo General Insurance Co., Ltd. (32.48%), Star Health and Allied Insurance Co., Ltd. (32.53%), and Sampoorna Suraksha (40.26%) reflected high OOPE percentages, while others like TATA AIG General Insurance Co., Ltd. (0%), Ecron Acunova Ltd. (3.13%), and Future Generali India Insurance Co., Ltd. (0.28%) provided near-complete coverage. These results reinforce existing research indicating that lack of insurance contributes significantly to financial hardship among patients [22,23].

High OOPE remains a major impediment to realizing universal health coverage and ensuring equitable access to quality healthcare services [24]. The significant variation in OOPE among privately insured patients suggests underlying issues within private health insurance benefit structures. These may include restricted coverage, low claim ceilings, narrow provider networks, or complex policy terms that are not well understood by policyholders. Moreover, cost-sharing mechanisms such as co-payments and deductibles commonly embedded in private plans contribute to higher OOPE. Compared to the comprehensive coverage under AB-PMJAY, these limitations weaken the financial protection provided by private insurance. This is consistent with the findings that publicly funded health insurance schemes are more effective in reducing OOPE than private alternatives, particularly for low-income populations [25]. Uninsured patients faced the heaviest financial burden, with a median OOPE of INR 66,929, accounting for 100% of their healthcare costs. This level of expenditure is particularly alarming, considering the economic vulnerabilities exacerbated during the pandemic. High OOPE has been associated with increased risk of impoverishment and delayed care-seeking, and these findings are consistent with multiple studies that flagged OOPE as a leading cause of health-related financial distress in India [11].

Integrating diagnosis-related groups (DRGs) into AB-PMJAY has the potential to minimize OOPE by introducing standardized reimbursement rates for specific diagnoses or procedures. This model can reduce cost variability across hospitals and insurers, which our findings suggest is a key driver of OOPE disparities, particularly within private insurance. By promoting transparent, predictable pricing, DRGs may also reduce patient confusion and prevent unexpected charges. Moreover, they incentivize hospitals to operate within fixed cost structures, which could discourage unnecessary services and improve resource efficiency. These benefits align with India’s goals of strengthening financial protection while controlling rising healthcare costs. Nonetheless, implementing DRGs comes with operational challenges, particularly in defining appropriate reimbursement rates and ensuring consistent enforcement across diverse healthcare settings in both public and private sectors [26,27]. The National Health Authority has initiated a pilot rollout of the DRG system in five states, namely Chhattisgarh, Haryana, Kerala, Maharashtra, and Meghalaya, and ABPMJAY would be the first Indian insurance program to adopt this payment model [19,28].

## 6. Conclusions

The COVID-19 pandemic has revealed critical gaps in India’s health financing framework. This study emphasizes the strong financial protection provided by AB-PMJAY, while also exposing the limitations of private health insurance in shielding patients from substantial healthcare costs. As the country progresses toward universal health coverage, there is a pressing need to expand public health insurance schemes that are inclusive, equitable, and effectively implemented. Additionally, strengthening regulation and accountability in the private insurance sector is essential. Although this study does not assess regulatory effectiveness, the observed inconsistencies in OOPE among private insurers suggest a potential role for improved regulation and accountability. Future research should examine how regulatory interventions impact financial protection. While AB-PMJAY is designed as a fully publicly funded scheme with zero OOPE, this study reaffirms its real-world implementation success in ensuring patients experienced no additional costs. This indicates effective operationalization of the scheme during a high-demand period like the COVID-19 pandemic. Although private insurance reduced OOPE compared to being uninsured, patients still faced considerable expenses. The stark difference in OOPE of 100% for uninsured patients, 21.16% for privately insured, and 0% for AB-PMJAY beneficiaries underscores the importance of further expanding AB-PMJAY to reach all vulnerable populations. This study did not capture household preferences or motivations for choosing public versus private care during the pandemic. However, previous research suggests that factors such as perceived quality, availability of services, and urgent care needs may have influenced the preference for private healthcare, thereby contributing to higher OOPE [29]. Future research should explore how such behavioral and system-level drivers affected healthcare utilization during the COVID-19 period. While the findings provide valuable insights, they should be interpreted with caution when generalizing to all healthcare settings in India. Future research should explore OOPE using multi-center data, incorporate outpatient and death-related cases, and apply marginal or comparative analyses. Evaluating the implementation of diagnosis-related groups (DRGs) under AB-PMJAY could also help assess standardization efforts aimed at reducing OOPE and improving transparency. An important limitation of this study is the absence of detailed diagnostic categorization to distinguish between admissions directly caused by COVID-19 and those due to comorbid or unrelated conditions in patients who tested positive. This could partially explain the observed variation in OOPE across insurance providers, as the nature and intensity of care required may have differed.

## Figures and Tables

**Table 1:** Distribution of cases across various categories.

Patient Category	No. of Cases	Percentage
ABPMJAY	1161	42.76%
Private Health Insurance	606	22.32%
Uninsured Patients	948	34.91%
Total	2715	100

**Table 2:** Number of cases, median and mean billing amount, OOPE as average percentage (%) of billing amount, median, and mean OOPE.

Patient Category	No. of Cases	Total Percentage of Cases (%)	Median Billing Amount/Mean Billing Amount (INR)	OOPE as an Average Percentage (%) of the Billing Amount	Median OOPE/Mean OOPE (INR)
ABPMJAY	1161	43	126,260 * (USD 1694.40)	0	0
Private Health Insurance	606	22	92,938 * (USD 1247.22)	21.16	19,728 *** (USD 264.75)
Uninsured Patients	948	35	66,929 * (USD 898.18)	100	66,929 *** (USD 898.18)

* The median billing amount was used as a measure of central tendency in cases where the Shapiro–Wilk test yielded a *p*-value less than 0.05, indicating a significant deviation from normality in the data distribution. *** Median OOPE was used to measure central tendency when the Shapiro–Wilk test *p*-value was less than 0.05, indicating a significant deviation from normality.

**Table 3:** Private health insurance: average OOPE as a percentage of the billing amount.

Private Health Insurance	No. of Cases	Total Percentage of Cases (%)	Median Billing Amount/Mean Billing Amount (INR)	Average OOPE as a Percentage (%) of the Billing Amount	Median/Mean OOPE
Future Generali India Insurance	1	0.03	146,496 ** (USD 1966)	0.28	415 ****
Good Health Insurance TPA	1	0.03	5977 ** (USD 80)	25.84	1545 ****
IFFCO-Tokio General Insurance	1	0.03	115,883 ** (USD 1555)	22.22	25,750 ****
Reliance General Insurance	1	0.03	72,426 ** (USD 971.95)	21.80	15,784 ****
Tata AIG General Insurance	1	0.03	141,805 ** (USD 1903)	0.0	0 ****
Vipul Medcorp TPA	1	0.03	267,885 ** (USD 3595)	4.05	10,870 ****
Aditya Birla Health Insurance	2	0.09	46,150 ** (USD 619.33)	9.78	4515 ****
Health India	2	0.09	254,460 ** (USD 3415)	3.96	10,084 ****
Ecron Acunova	3	0.13	19,769 ** (USD 265.30)	0	0
Apollo Munich Health Insurance	3	0.13	41,004 ** (USD 550.27)	7.39	3032 ****
Care Health Insurance Company	3	0.13	120,878 ** (USD 1622)	14.81	17,898 ****
United Healthcare India	3	0.13	86,576 ** (USD 1162)	21.91	18,969 ****
Universal Sompo General Insurance	3	0.13	238,099 **** (USD 3195.3)	32.48	77,330
Religare Health Insurance	5	0.18	96,553 (USD 1296)	24.33	23,489
Bajaj Allianz General Insurance	6	0.22	105,474 ** (USD 1416)	10.60	11,181 ***
HDFC	6	0.22	62,332 ** (USD 836.49)	12.50	7789 ****
ICICI Lombard General Insurance	6	0.22	119,793 ** (USD 1608)	5.56	6667 ****
Paramount Health Services Insurance TPA	6	0.22	106,817 ** (USD 1434)	26.55	28,362 ***
United Healthcare India	8	0.062	146,374 ** (USD 1964)	18.69	27,369 ****
MDINDIA Health Insurance TPA	15	0.55	143,916 ** (USD 1931)	18.90	27,198 ****
Sampoorna Suraksha	29	1.06	60,819 ** (USD 816.19)	40.26	24,488 ***
Vidal Health TPA	37	1.36	109,275 ** (USD 1467)	26.42	28,874 ****
Star Health and Allied Insurance	50	1.84	144,049 * (USD 1933)	32.53	46,855 ***
Medi Assist India TPA	99	3.65	121,473 ** (USD 1630)	21.98	26,697 ***
Medicare	314	11.57	70,814 ** (USD 950.32)	16.58	11,743 ***
Total	606	22.16%		17.03	

* Median billing was used to measure central tendency when the Shapiro–Wilk test *p*-value was less than 0.05, indicating a significant deviation from normality (e.g., COVID-19 cases under AB-PMJAY). ** Mean billing was used to measure central tendency when the Shapiro–Wilk test *p*-value exceeded 0.05, indicating a normal distribution. *** Median OOPE was used to measure central tendency when the Shapiro–Wilk test *p*-value was less than 0.05, indicating a significant deviation from normal distribution. **** Mean OOPE was used when the Shapiro–Wilk test *p*-value exceeded 0.05, indicating a normal distribution.

## Data Availability

The original contributions presented in this study are included in the article. Further inquiries can be directed to the corresponding author(s).
